# Desmoplastic Fibroma of the Mandible in a 10-Year-Old Female Patient: A Case Report

**DOI:** 10.7759/cureus.106697

**Published:** 2026-04-09

**Authors:** Olga Bellou, Fani Pitsigavdaki, Evangelos Kostares, Eleni Chra, Ourania Schoinohoriti

**Affiliations:** 1 University Department of Oral and Maxillofacial Surgery, General Children’s Hospital of Athens “Panagiotis and Aglaia Kyriakou”, Athens, GRC; 2 Department of Oral and Maxillofacial Surgery, School of Dentistry, National and Kapodistrian University of Athens, Athens, GRC; 3 University Department of Oral and Maxilofacial Surgery, Evangelismos General Hospital, Athens, GRC; 4 Department of Anatomical Pathology, General Children’s Hospital of Athens “Panagiotis and Aglaia Kyriakou”, Athens, GRC

**Keywords:** benign, case report, desmoplastic fibroma, locally aggressive, mandible, mandible swelling

## Abstract

Desmoplastic fibroma (DF) is an extremely rare benign intraosseous tumor of mesenchymal origin that exhibits locally aggressive behavior with a high recurrence rate. Although mainly affecting patients between the second and third decades of life, it rarely involves the jaw bones, with the mandible being the most affected. Complete surgical removal of DF may require curettage or resection of the surrounding bone to prevent recurrence. This article reports the case of a 10-year-old girl who was referred to our department by a dentist for the evaluation of a painless swelling at the left angle of the mandible. Following radiographic examination (orthopantomogram and CT scan) and incisional biopsy, the patient was successfully submitted to tumor excision and curettage of the adjacent mandibular bone. Complete osseous healing was confirmed on the patient’s follow-up examination one year postoperatively. Although DF of the jaws represents a diagnostic (mimicking a variety of fibro-osseous lesions) and therapeutic (due to its locally aggressive behavior) challenge, early detection, corroborated by histopathological examination, along with prompt surgical removal within clear margins, may ensure minimal recurrence.

## Introduction

According to the World Health Organization (WHO), desmoplastic fibroma (DF) is a rare benign bone tumor that occasionally exhibits locally aggressive behavior. Histologically, it consists of spindle cells, set in large amounts of collagen, resembling desmoid-type fibromatosis [1]. Jaffe described it first in 1958 as a tumor affecting various sites (scapula, tibia, and femur) [2], while Griffith and Irby were the first to report in 1965 a case of DF involving the jaws [3]. Although sporadically described, DF represents only 0.1 % of all jawbone tumors [4,5].

Among its various clinical findings, pain is the most frequent, followed by swelling and facial asymmetry; tooth displacement and/or mobility and limited mouth opening are less common. Imaging usually reveals a well-defined uni- or multi-locular radiolucency, causing jaw expansion [6].

The diagnosis of DF is challenging due to its resemblance to a wide variety of benign or malignant tumors with similar clinical, radiological, and histopathological findings. Given the rarity of DF, knowledge regarding its prognosis, clinical course, and recurrence patterns is primarily based on case reports or series.

Following the CARE statement guidelines, we present an interesting case of DF in a 10-year-old girl that was excised under general anesthesia in our department. This case report contributes to the limited available literature, emphasizing the need for further investigation regarding both the diagnostic approach and the treatment protocols.

## Case presentation

A 10-year-old girl was referred to the Department of Oral and Maxillofacial Surgery of the General Children’s Hospital of Athens “Panagiotis and Aglaia Kyriakou” by a general dentist for further evaluation of a four-month painless swelling at the left mandibular angle, detected six months earlier during a routine dental examination.

**Figure 1:**
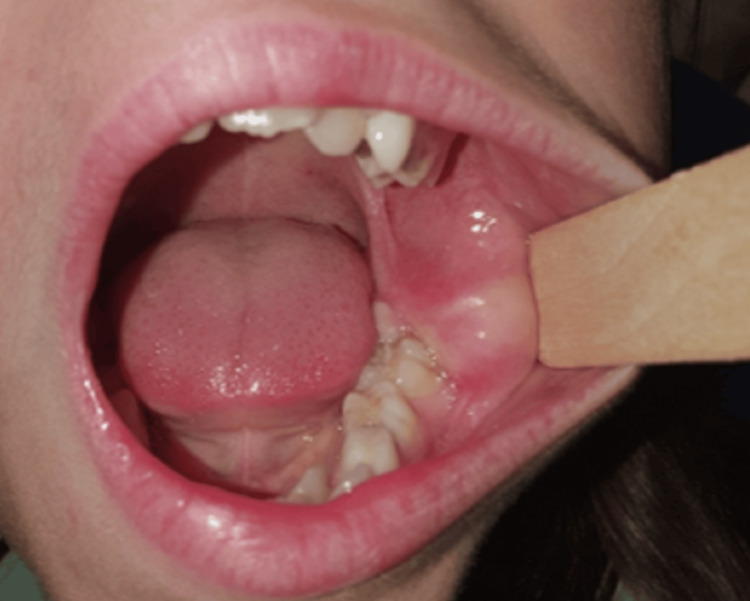
Initial intraoral image of the patient showing a swelling at the left mandibular angle and ramus.

The patient mostly complained of a painless, gradually progressing for four months swelling of hard consistency at the left mandibular angle that caused moderate asymmetry of the lower face, without affecting the overlying mucosa or impairing the function of the inferior alveolar nerve (Figure 1). No history of trauma or infection was recorded. Laboratory findings were within normal limits.

**Figure 2:**
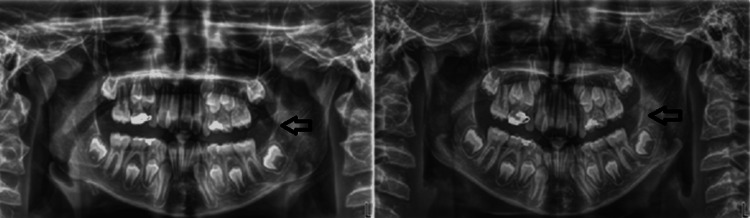
Preoperative orthopantomograms of the patient obtained with a three-month interval showing a relatively well-defined radiolucency at the left mandibular angle and ramus.

**Figure 3:**
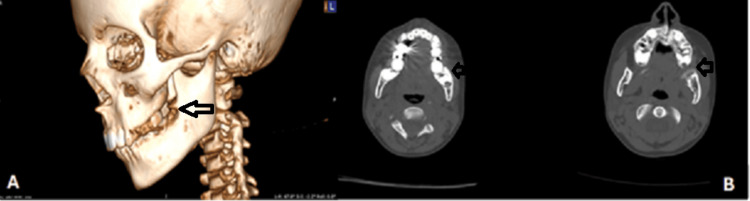
Preoperative CT scan of the patient. (A) Three-dimensional reconstruction showing the lesion adjacent to tooth #37. (B) Axial sections showing discontinuity of the buccal cortex at the left side of the mandible.

Initial imaging, including orthopantomograms and a CT scan of the facial skull, demonstrated a well-defined radiolucency, extending from tooth #36 to the mandibular ramus, causing expansion of the left mandibular angle (Figures 2, 3).

Based on the clinical and radiographic findings of the lesion, differential diagnosis included primarily benign lesions such as odontogenic tumors (e.g., ameloblastoma, odontogenic myxoma), aneurysmal bone cyst, fibrous dysplasia, osteomyelitis, intraosseous hemangioma, central giant cell tumor, central ossifying fibroma, non-ossifying fibroma, odontogenic keratocyst, and desmoid tumor, as well as secondary malignancies, such as low-grade osteosarcoma, low-grade fibrosarcoma, and Ewing sarcoma.

An incisional biopsy, performed under general anesthesia, showed numerous spindle cells within an abundant collagenous stroma. These histological findings were consistent with the diagnosis of intraosseous DF, a benign but locally aggressive fibroblastic tumor of the bone.

Surgical excision was decided as the optimal therapeutic modality and was performed two weeks after the incisional biopsy. Under general anesthesia, the tumor was exposed through an intraoral incision, extending from the ramus to the pterygomandibular raphe. The inferior alveolar neurovascular bundle was identified and protected. The tumor, measuring approximately 2 x 1.5 cm, was completely excised along with the involved tooth bud #37 to achieve macroscopically clear margins. Given that the tumor involved the buccal cortex, thorough curettage of the surrounding bone was subsequently performed to minimize the risk of residual disease and recurrence. The excised specimen was sent for histopathologic examination, which corroborated the diagnosis of the incisional biopsy.

**Figure 4:**
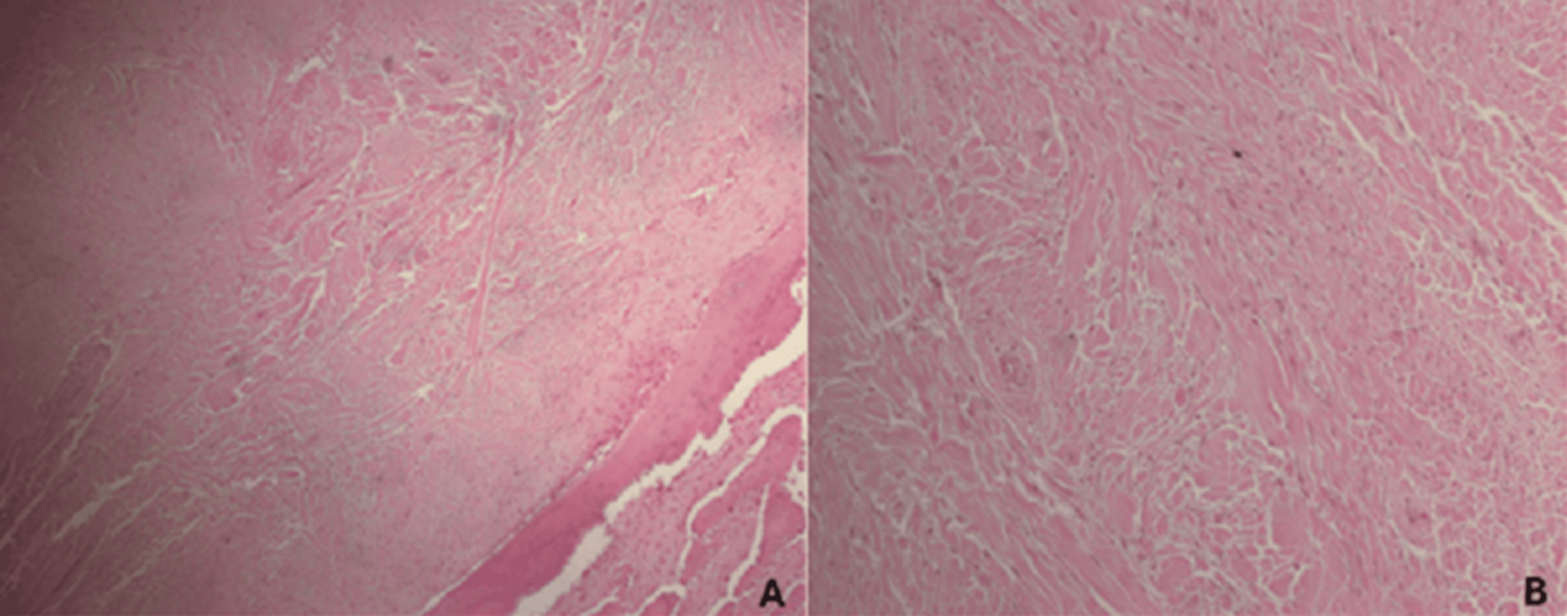
Microphotographs of the excised specimen revealing a poorly demarcated tumor mass composed of bland spindle or stellate fibroblasts with no or minimal mitotic activity, separated by abundant collagenous stroma; no pleomorphism, cellular atypia, or necrosis were observed. (A) Magnification ×40. (B) Magnification ×100.

According to the histopathology report, the lesion consisted of numerous spindle cells and cells with ovoid nuclei, set within abundant collagen with varying degrees of myxoid changes, reminiscent of desmoid-type fibromatosis. Rare mitotic activity was registered, without signs of cellular atypia or necrosis (Figure 4). On the periphery of the sample, bony particles of the mandible with hemorrhagic infiltration and necrotic trabeculae were observed. Immunohistochemistry revealed Vimentin (+), SMA (+), B-catenin (+), Desmin (-), S100 (-), KerAE1/AE3 (-), CD34 (-), h-Caldesmon (-), HHF-35 (-), muc-4 (-), CDK4 (-), and cell proliferation index Ki-67 of 1-2%.

The patient was discharged from the hospital on the second postoperative day, and her parents were instructed to maintain her on a semi-solid diet for approximately one month. The postoperative follow-up protocol included clinical examinations at one month and every three months thereafter for the first year. Radiographic evaluation via orthopantomogram was performed at one month postoperatively and subsequently at six-month intervals, including a CT scan at the one-year follow-up, to confirm complete osseous healing.

**Figure 5:**
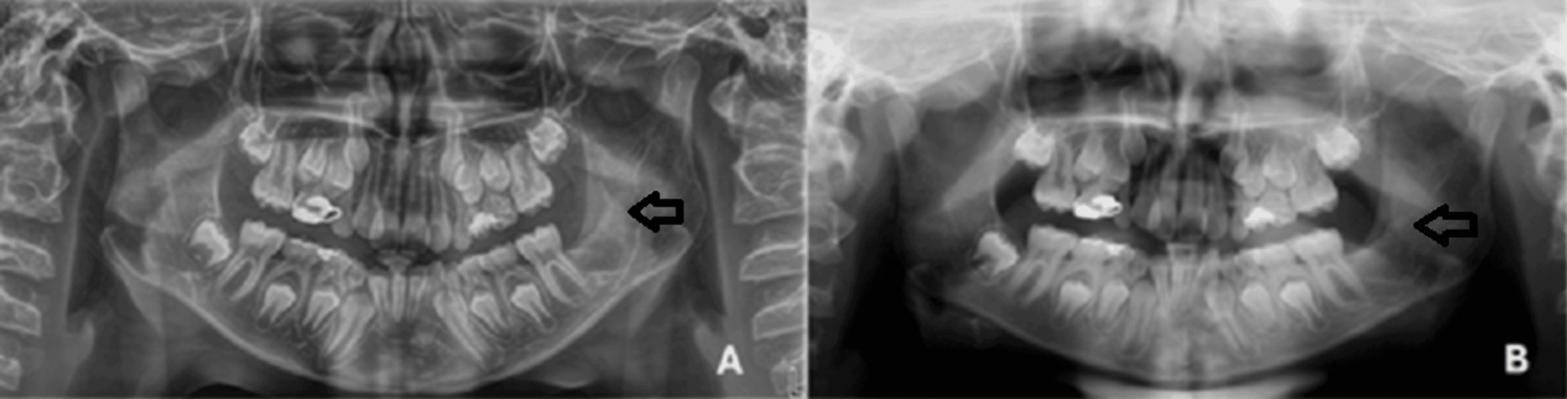
Postoperative orthopantomograms of the patient (A) one-month postoperatively and (B) at the one-year follow-up examination demonstrating significant bone remodeling of the defect, without evidence of recurrence.

Both clinically and radiographically, the healing of the defect has been uneventful, and no signs of residual disease or recurrence have been registered so far (Figure 5). Hypoesthesia of the left half of the lower lip that had been reported immediately following surgery resolved spontaneously six months postoperatively.

## Discussion

DF is a rare, slowly progressing, benign but locally aggressive bone tumor, affecting primarily the femur (15%), pelvis (13%), radius (12%), and tibia (9%) [7]. The jaws are rarely affected, with a clear predilection for the mandible [8].

According to the WHO, DF represents a locally infiltrative fibrogenic neoplasm, composed of well-differentiated bland spindle cells, set in abundant collagen, reminiscent of desmoid-type fibromatosis, with an incidence of 0.1% among bone tumors [4,9]. Although it is considered the intraosseous counterpart of desmoid-type fibromatosis, recent studies have failed to identify mutations in exon 3 of *CTNNB1* that encodes B-catenin, thus clearly differentiating DF from desmoid-type fibromatosis on a genetic basis [10]. Regarding the etiology of DF, several potential causes have been suggested, such as trauma, endocrine disorders, or genetic predisposition [11].

According to the systematic review by Loureiro et al., DF of the jaws occurs predominantly in women (female-to-male ratio of 1.26: 1) at a mean age of 18.2 years (with an age range from 6 months to 66 years) and affects most commonly the posterior mandible [6].

DF usually appears as a painless swelling of the jaw that causes facial asymmetry [7] and, less commonly, tooth displacement or mobility, toothache, root divergence or resorption, limited mouth opening/trismus with or without malocclusion, intraoral ulcerations, and temporomandibular disorders [5,12,13]. Signs of infection may be present, with or without enlarged peripheral lymph node(s) or elevated ear lobe on the involved side [14]. Pathological fractures have also been reported [11]. Impacted teeth may influence the detection and management of DF, whereas their presence may affect the eruption and position of permanent dentition, thus warranting early detection, accurate diagnosis, and careful surgical planning [6].

The radiographic features of DF are non-specific. It is usually depicted as a well-delineated uni- or multi-locular radiolucency and, less commonly, as a radiopaque or mixed lesion with poorly demarcated borders that may cause expansion and thinning of the cortical plates [6,15]. Rapid cortical expansion and perforation make it difficult to determine whether the lesion is a DF located primarily at the jaw and extending to soft tissues or a soft tissue fibromatosis extending into the bone [16].

According to Karimi et al., DF may appear as a lobulated lesion with small, rounded segments, creating a soap bubble pattern radiographically [7]. Periosteal reaction is a rare radiographic finding of DF, producing a sunray pattern that may lead to its misdiagnosis as osteosarcoma, especially when combined with rapid growth and extended bone destruction [14,16].

An orthopantomogram is typically obtained for the initial radiographic evaluation of DF in the jaws. Depending on the cortical involvement and extraosseous extent of the lesion, further imaging analysis is required, through a CT scan or an MRI [4]. MRI is most valuable for separating intraosseous tumors from normal bone marrow to assist in surgical planning, whereas CT is recommended to demonstrate cortical breakthrough [14].

Histology is considered the gold standard for the diagnosis of DF. Among the assorted histological features of DF, the presence of mature fibrous stroma and areas with abundant collagen fibers oriented in a linear or twirling arrangement that separate the marginally atypical spindle-shaped fibroblasts with round or fusiform nuclei are the most common [7,12]. No signs of cellular atypia, necrosis, mitotic activity, or nuclear hyperchromatism are observed [13,16]. Myxoid connective tissue and hyalinized areas may be present; areas with cellular hypercellularity or pleomorphism may rarely be observed, with the latter playing a major role in recurrence [6,16]. The lack of capsule and infiltrative nature also represent typical histologic features of DF [17]. Moreover, DF may appear as a manifestation of other conditions, such as familial adenomatous polyposis or tuberous sclerosis [18,19].

Biopsy should be taken from the core of the lesion, as reactive bone consistently present at its periphery may produce the illusion of new bone formation and lead to the misdiagnosis of a fibro-osseous lesion (fibrous dysplasia, myofibroma, odontogenic fibroma, and cemento-ossifying fibroma) or malignancies, such as low-grade fibrosarcoma or low-grade intraosseous sarcoma [16,17]. The absence of nuclear pleomorphism, the low mitotic activity, the lack of odontogenic epithelium, and the lack of cementum or bone components are helpful to histologically differentiate DF from other jaw lesions [4].

The immunomarkers associated with a positive staining for DF include Vimentin, SMA, p53, reticulin, and β-catenin. Hauben et al emphasized the involvement of the APC/β-catenin signaling pathway in the development of DF, indicating that alterations in the expression of β-catenin may inactivate the *APC* gene or activate *CTNNB1* mutations [10]. However, the poor strand of DNA from decalcified sections has been an obstacle in this analysis [15]. The stainings for S100, keratin, and histiocytic markers are negative [5,12].

Different treatment modalities for the DF have been described over the years, including surgical curettage, segmental resection, en bloc resection with wide margins followed by reconstruction, neoadjuvant and/or adjuvant cytotoxic radiotherapy, neoadjuvant and/or adjuvant cytotoxic chemotherapy, and adjuvant hormone therapy [7,12,14]. Although Zainuddin et al described a case of DF treated with enucleation without recurrence [20], the contemporary benchmark approach consists of complete surgical removal within healthy margins. Adjuvant radiotherapy is indicated as a supplementary treatment approach when wide surgical excision has left positive margins or led to recurrence [12].

Recurrence is a common complication mainly at the posterior mandible that is directly linked with the implemented surgical technique; the reported recurrence rates following excision and enucleation range from 20% to 40%, thus highlighting the role of a proper surgical protocol, consisting of complete removal and the need for long-term postoperative follow-up [14,20]. In this case, the follow-up period was only one year postoperatively, but the patient will hopefully remain under follow-up at least for the next five years or until growth is completed.

As this is a single case report, the findings cannot be generalized and do not allow firm conclusions regarding optimal diagnostic algorithms or management strategies of DF. In addition, the relatively short follow-up period limits the assessment of long-term recurrence risk, which has been recorded in the pertinent literature.

## Conclusions

DF is a benign lesion with locally aggressive behavior that rarely affects the jaws of adolescents and young adults. Its diagnosis requires the exclusion of various benign and malignant lesions. Various imaging modalities, combined with incisional biopsy, immunohistochemical, and molecular analysis, are crucial not only for diagnosis but also for optimal treatment planning. Although the presented case raises awareness of the challenging diagnosis and management of DF of the jaws and emphasizes the importance of a multidisciplinary treatment approach, the findings cannot be generalized and do not allow firm conclusions regarding optimal diagnostic algorithms or management strategies. In addition, the relatively short follow-up period limits assessment of long-term recurrence risk.

## References

[1] Choi JH, Ro JY (2021). The 2020 WHO Classification of Tumors of Bone: an updated review. Adv Anat Pathol.

[2] Jaffe HL (1958). Tumors and Tumorous Conditions of the Bones and Joints.

[3] Griffith JG, Irby WB (1965). Desmoplastic fibroma. Report of a rare tumor of the oral structures. Oral Surg Oral Med Oral Pathol.

[4] Woods TR, Cohen DM, Islam MN, Rawal Y, Bhattacharyya I (2015). Desmoplastic fibroma of the mandible: a series of three cases and review of literature. Head Neck Pathol.

[5] Segard T, Bertin H, Lepine C, Guyonvarc'h P (2024). Desmoplastic fibroma of the jaw: a case report and review of the literature. J Stomatol Oral Maxillofac Surg.

[6] Loureiro FJ, Nogueira WR, Dutra MJ (2025). Desmoplastic fibroma of the gnathic bones-a systematic review. Oral Dis.

[7] Karimi A, Derakhshan S, Moradzadeh Khiavi M, Mosavat F, Mirjalili F (2020). Desmoplastic fibroma of the jaws: a case series and review of literature. Iran J Pathol.

[8] Siddiqui HK, Khan SA, Aijaz A, Qureshi MB (2024). Unraveling the challenges in the diagnosis and management of desmoplastic fibroma of the mandible-a case report. BMC Oral Health.

[9] WHO Classification of Tumours Editorial Board (2022). WHO Classification of Tumours Series. Head and Neck Tumours.

[10] Hauben EI, Jundt G, Cleton-Jansen AM, Yavas A, Kroon HM, Van Marck E, Hogendoorn PC (2005). Desmoplastic fibroma of bone: an immunohistochemical study including beta-catenin expression and mutational analysis for beta-catenin. Hum Pathol.

[11] Titidej A, Jolehar M (2020). Rare desmoplastic fibroma in ramus and mandibular angle: presentation of a case report with review of literature. Acta Med Iranica.

[12] Al-Khateeb TH (2025). Mandibular versus maxillary desmoplastic fibroma: a pooled analysis of world literature and report of a new case. Eur J Dent.

[13] Kahraman D, Karakoyunlu B, Karagece U, Ertas U, Gunhan O (2021). Desmoplastic fibroma of the jaw bones: a series of twenty-two cases. J Bone Oncol.

[14] Said-Al-Naief N, Fernandes R, Louis P, Bell W, Siegal GP (2006). Desmoplastic fibroma of the jaw: a case report and review of literature. Oral Surg Oral Med Oral Pathol Oral Radiol Endod.

[15] Madakshira MG, Bal A, Verma RK (2019). Desmoplastic fibroma of the mandible: a rare gnathic bone tumor with a review of the literature. Autops Case Rep.

[16] Nisha S, Chetana C, Ranjini K, Adarsh K (2021). Desmoplastic fibroma of the mandible with unusual histopathological features. Indian J Pathol Microbiol.

[17] Motevasseli S, Yousefi Z, Dalili Kajan Z, Modanlou R, Roudbari N (2022). Periosteal reaction as a crucial radiographic finding for desmoplastic fibroma of the jaw bone in children: a case report. Imaging Sci Dent.

[18] Espinoza-Coronado AM, Loyola-Rodríguez JP, Olvera-Delgado JH, García-Cortes JO, Reyes-Macías JF (2018). Desmoplastic fibroma recurrence associated with tuberous sclerosis in a young patient. Case Rep Dent.

[19] Andrade N, Sharma S, Gupta V, Desai R, Palve S (2024). Desmoplastic fibroma of the mandible in a 5-year-old child as an early oral manifestation of familial adenomatous polyposis. Int J Oral Maxillofac Surg.

[20] Zainuddin NI, Chin Kai L, Lim D, Wm T (2023). Desmoplastic fibroma of the mandible: a case without recurrence after enucleation. Cureus.

